# Influence of implant macrodesign and insertion load during implant placement on primary stability in artificial bone

**DOI:** 10.1186/s40729-025-00644-4

**Published:** 2025-08-24

**Authors:** Yiwen Wang, Yoko Yamaguchi, Poyuan Hsueh, Daisuke Higuchi

**Affiliations:** 1 Department of Oral and Maxillofacial Biology, Evaluation of Orofacial Function, Graduate School of Oral Medicine, Matsumoto Dental University, 1780 Gobara, Hirooka, Shiojiri, Nagano, 399-0781 Japan; 2 Department of Prosthodontics, School of Dentistry, Matsumoto Dental University, 1780 Gobara, Hirooka, Shiojiri, Nagano, 399-0781 Japan; 3 Tokyo Clinic, Matsumoto Dental University, 9F Hulic&New GINZA8, 8-9-7 Ginza, Chuo-Ku, Tokyo, 104-0061 Japan

**Keywords:** Dental implant, Primary stability, Insertion torque value, Removal torque value, Insertion load, Artificial bone, Implant placement, Implant design, Low-density bone

## Abstract

**Background:**

Implant placement is a critical step for achieving primary stability. During this process, a compressive force, referred to as an “insertion load,” is applied through a handpiece or manual driver. However, the influence of the insertion load has not been quantitatively investigated. Since primary stability is essential for predicting successful osseointegration, clarifying the effects of the insertion load may contribute to improving the safety and success of implant treatment.

**Methods:**

Straight single-thread implants with pitches of 0.6 mm (S06) and 1.2 mm (S12) and double-thread implants with a 0.6 mm pitch (D06) were used (10 each). Insertion loads of the minimum required load, 5 N, 10 N, and 15 N were applied. Insertion torque values (ITVs), removal torque values (RTVs), and insertion times were measured (*p* = 0.05). The interface between the implants and the simulated bone was observed with a digital microscope under each load.

**Results:**

The minimum insertion load was greatest in the order of S12 < S06 < D06. Variation in the insertion load did not affect the ITV or RTV, but lower loads extended the insertion time. Increased insertion loads led to more voids caused by bone fragment loss, especially in the double-thread designs.

**Conclusions:**

While the insertion load did not directly influence the ITV or RTV, it varied by implant design. Higher loads potentially damage the surrounding bone, indicating the importance of careful load management.

## Background

Implant treatment is a method used to restore the ability to chew by attaching crowns or dentures to implants that are inserted into the jawbone. It is used for many types of cases where teeth are missing, including complete edentulism [1, 2], partial edentulism [2, 3], and single-tooth loss [2, 3]. Because of its high success rate [2, 4, 5], it is very predictable [1, 3, 6], and patients are generally very satisfied; thus, it is considered an effective solution for missing teeth.

Despite the high success rates, failure rates of 3–7% have been reported [7–9]. An implant is considered a failure when there are signs or symptoms that indicate that it needs to be removed [5, 8]. Failures are generally divided into early failure and late failure [7–10]. Early failure is the inability to achieve osseointegration before the connection of the dental prostheses [8–10], whereas late failure occurs when the osseointegration obtained after the dental prostheses set is not maintained [8–10]. Although implant failure is multifactorial and difficult to specify, early failure has been associated with impaired bone healing and reduced primary stability [7, 10, 11], occurring at a frequency 2–5 times greater than that of late failure [7, 8]. Primary stability depends on bone density, and early failures are known to be more common in the posterior maxillary molar region [8, 11, 12]. A review of the biological factors involved in implant failure indicates that low primary stability may induce micromovements of the implant, leading to the formation of fibrous tissue at the bone–implant interface and subsequent early loss [9, 11, 12]. As primary stability is a key indicator for achieving efficient secondary stability (biological stability), strategies to prevent early failure are advantageous for the long-term prognosis of implants.

Insufficient primary stability, which is one of the factors contributing to early failure, is influenced mainly by bone quality and quantity, implant design, and surgical method [11–13]. Furthermore, primary stability is affected by cracks generated during implant insertion; if the bone–implant process experiences significant displacement and rotation, the initially contacted surrounding bone may become overloaded, leading to further damage [11, 14]. Therefore, during implant insertion, it is imperative to simultaneously ensure sufficient mechanical engagement between the bone and the implant while minimizing damage to the surrounding bone. Clinically, implant insertion is performed using either a dental engine or a manual driver. Force needs to be applied either using a handpiece or manually to insert the implant into its socket, and accurately measuring this force is a significant challenge. Consequently, to understand the effect of the insertion load on primary stability, we conducted a simulation study with an emphasis on standardization and reproducibility.

## Methods

### Implants

**Fig. 1:**
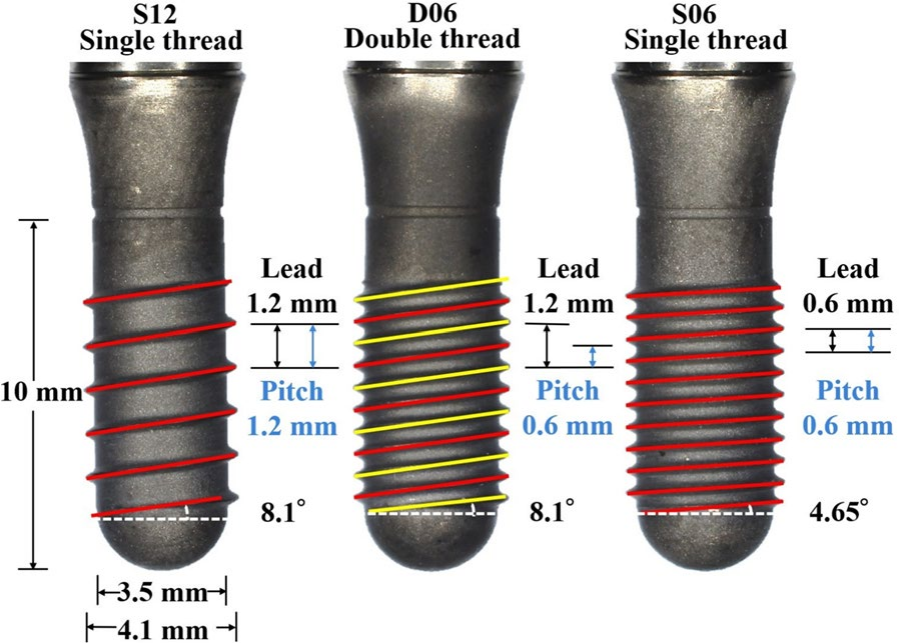
Implants used in the experiment

The prototype implants were fabricated from commercially pure titanium with a parallel design. Three types of designed implants with different pitch, lead, and helix angles were used: S12, S06, and D06. Ten implants of each type were used (Fig. 1). To eliminate the influence of diametral stresses on shear phenomena during insertion, the outer and inner diameters were made equal. Moreover, to eliminate the effect of frictional torque, the implant surfaces were machined, thereby eliminating the impact of surface roughness on the coefficient of friction.

### Preparation of implant sockets in artificial bone

**Fig. 2:**
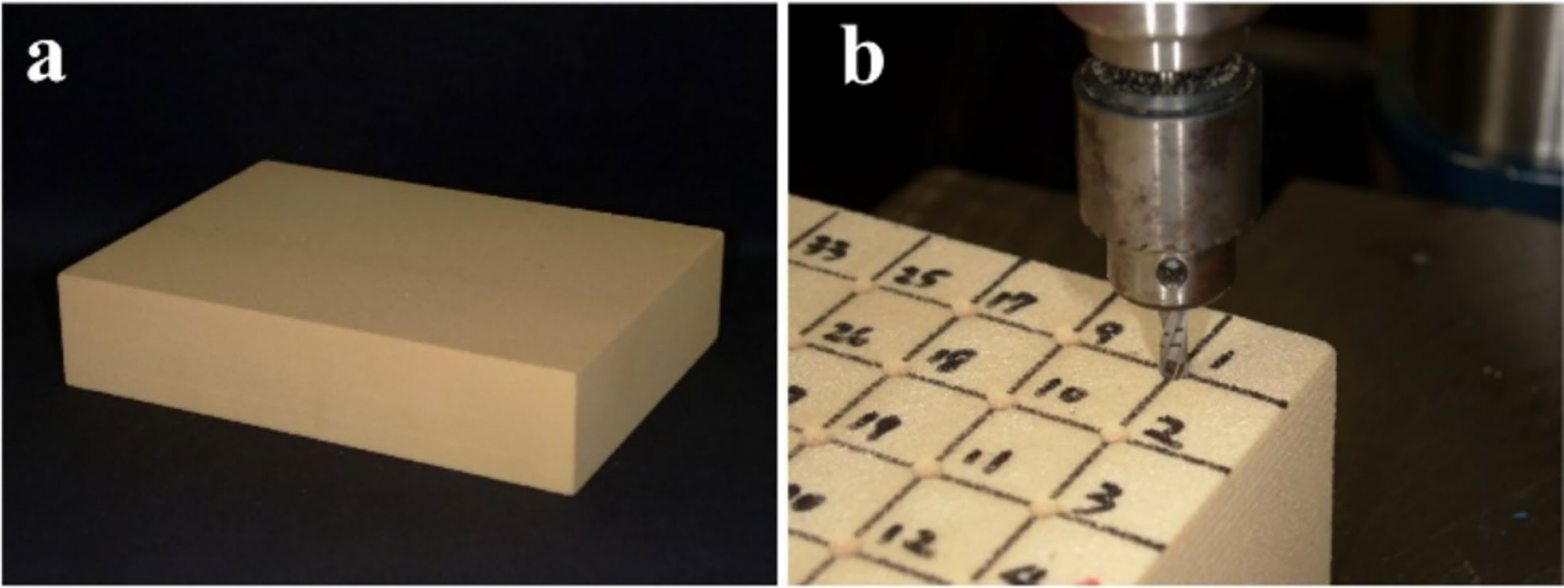
Artificial bone used in this study and preparation of the implant sockets. **a** The block is 18 cm × 4 cm × 13 cm in size, and its physical properties include a compressive strength of 8.4 MPa, a tensile strength of 5.6 MPa, and an elastic modulus of 284 MPa. **b** The implant sockets were prepared using a drilling machine at 15 mm intervals

The implant stability achieved through mechanical engagement during insertion is affected by the frictional conditions at the interface. However, previous studies have shown that the dynamic coefficient of friction in artificial bone does not differ significantly between dry and wet conditions [15]. Prototype implants [16] and rigid polyurethane foam [15–17], a substitute test medium for human cancellous bone used in comparative testing of orthopedic bone screws by the American Society for Testing and Materials [18], were employed. In this study, a homogeneous rigid polyurethane foam block that approximates the bone density of the maxillary molar region and lacks cortical bone (pcf20, Solid Rigid Polyurethane Foam, Sawbones, Pacific Research Laboratories, Washington, USA) was used (Fig. 2a). A drill press (DP-550SDI, digital drill press, PAOCK Corporation, Power Sonic, Tokyo, Japan) was used for sequential enlargement, resulting in a final osteotomy with a diameter of 3.5 mm and a depth of 10 mm (Fig. 2b).

### Insertion load

**Fig. 3:**
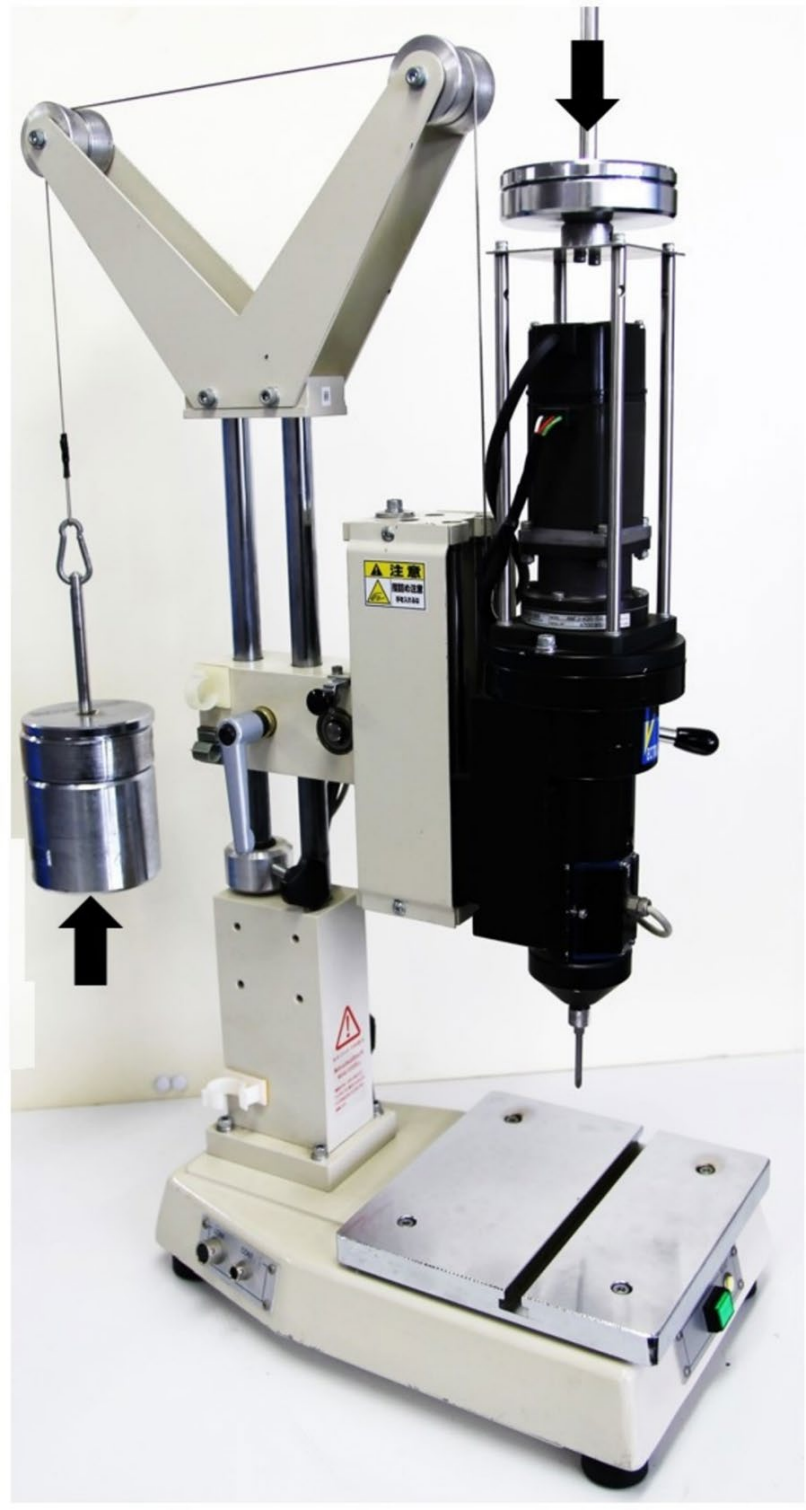
PC torque analyzer. The weight of the device attached to the torque analyzer was set to zero before the test using the thrust load adjustment weight (↑). When the implant was inserted, an insertion load (↓) equivalent to the placement load was used

The insertion loads were defined at four levels: the minimum insertion load required for each implant and fixed loads of 5.0 N, 10.0 N, and 15.0 N. To determine the minimum insertion load, a torque meter (PC Torque Analyzer TVRQ-5DRU, Vectrix, Tokyo, Japan) capable of sampling at 1-ms intervals was used (Fig. 3). Moreover, because the weight of the insertion apparatus can directly affect the insertion load, the weight of the insertion device on the torque analyzer was preset to zero using a balancing method (Fig. 3). The insertion load was applied perpendicular to the long axis of the implant. For each implant type (S12, S06, and D06), a 5.0 N weight was initially placed on the torque analyzer, and then the load was reduced in 0.5 N increments until all implants could be inserted. The minimum insertion load was defined as the smallest load at which all 10 implants could be successfully inserted on the basis of visual confirmation of proper implant insertion without slipping on the implant bed.

### Measurement of the insertion and removal torque values and insertion time

The insertion and removal torque values for each implant were measured using incremental Newton weights (brass with chrome plating, Taisho Scales Manufacturing Co., Ltd., Osaka, Japan) corresponding to the minimum insertion load, 5.0 N, 10.0 N, and 15.0 N. Torque–time curves were recorded continuously from the start of implant insertion until removal while the designated insertion load was maintained. During insertion, each implant was vertically aligned with the center axis of the implant bed and inserted into the implant bed at 15 rpm. To avoid postinsertion stress relaxation, the implant was immediately removed via reverse rotation. The maximum values on the insertion and removal torque–time curves were defined as the insertion torque value (ITV) and removal torque value (RTV), respectively. Furthermore, for each implant and each insertion load, the patterns of the torque–time curves were analyzed, and the time required to reach the ITV (insertion time) was used to assess the effect of the insertion load.

### Observations of the bone-implant interface

To observe the bone–implant interface, which cannot be adequately visualized with conventional resin-embedded sections, a split-block method was employed [19]. For each insertion load, split-block samples were prepared from each implant, and an optical digital microscope (HRX-01 digital microscope, Hirox, Tokyo, Japan) was used to count the number of voids. The voids are presumed to result from disruption of the artificial bone at the bone–implant interface. The total void area was then measured using image analysis software (ImageJ, National Institutes of Health, Maryland, USA).

### Statistical analysis

For each measured parameter, the mean and standard deviation were calculated. Two-way analysis of variance (ANOVA) with implant type and insertion load as factors was performed, followed by Tukey’s multiple comparisons test (JMP4, SAS Institute, Tokyo, Japan) (*p* < 0.05).

## Results

### Minimum insertion load

**Fig. 4:**
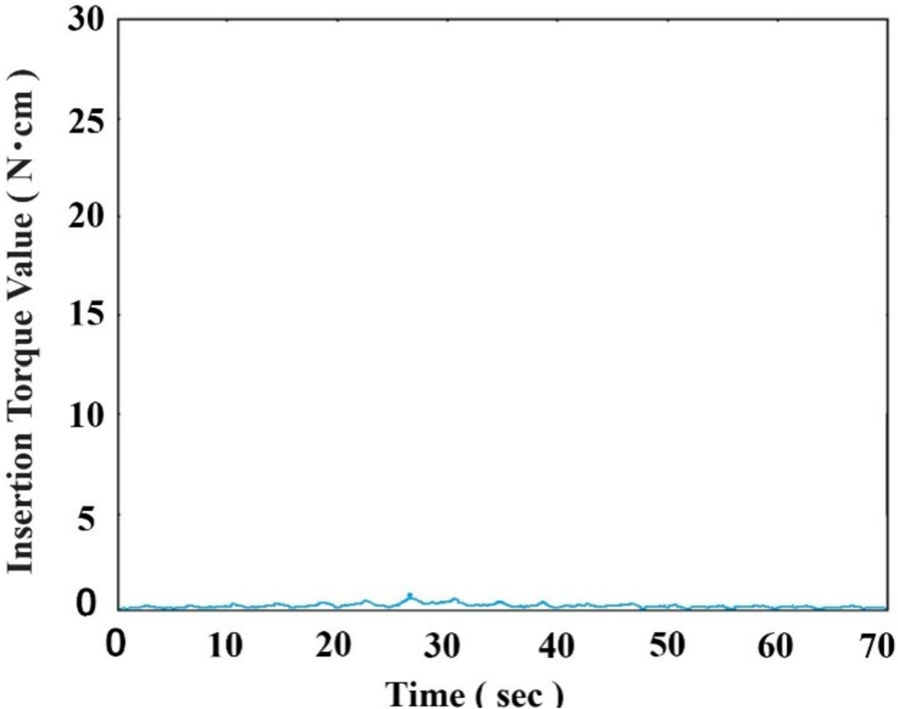
An example of a torque‒time curve of the blue line on an implant under free-spinning conditions for placement

During the measurement process, when the insertion load was gradually reduced in 0.5 N increments, a threshold was reached beyond which either implant insertion could no longer be accomplished or the implant exhibited slippage at the entrance of the bone despite maintaining the predetermined rotational speed (Fig. 4). The minimum insertion loads were 2.0 N for S12, 3.5 N for S06, and 4.0 N for D06, with significant differences among them (*p* < 0.05).

### Torque values

**Fig. 5:**
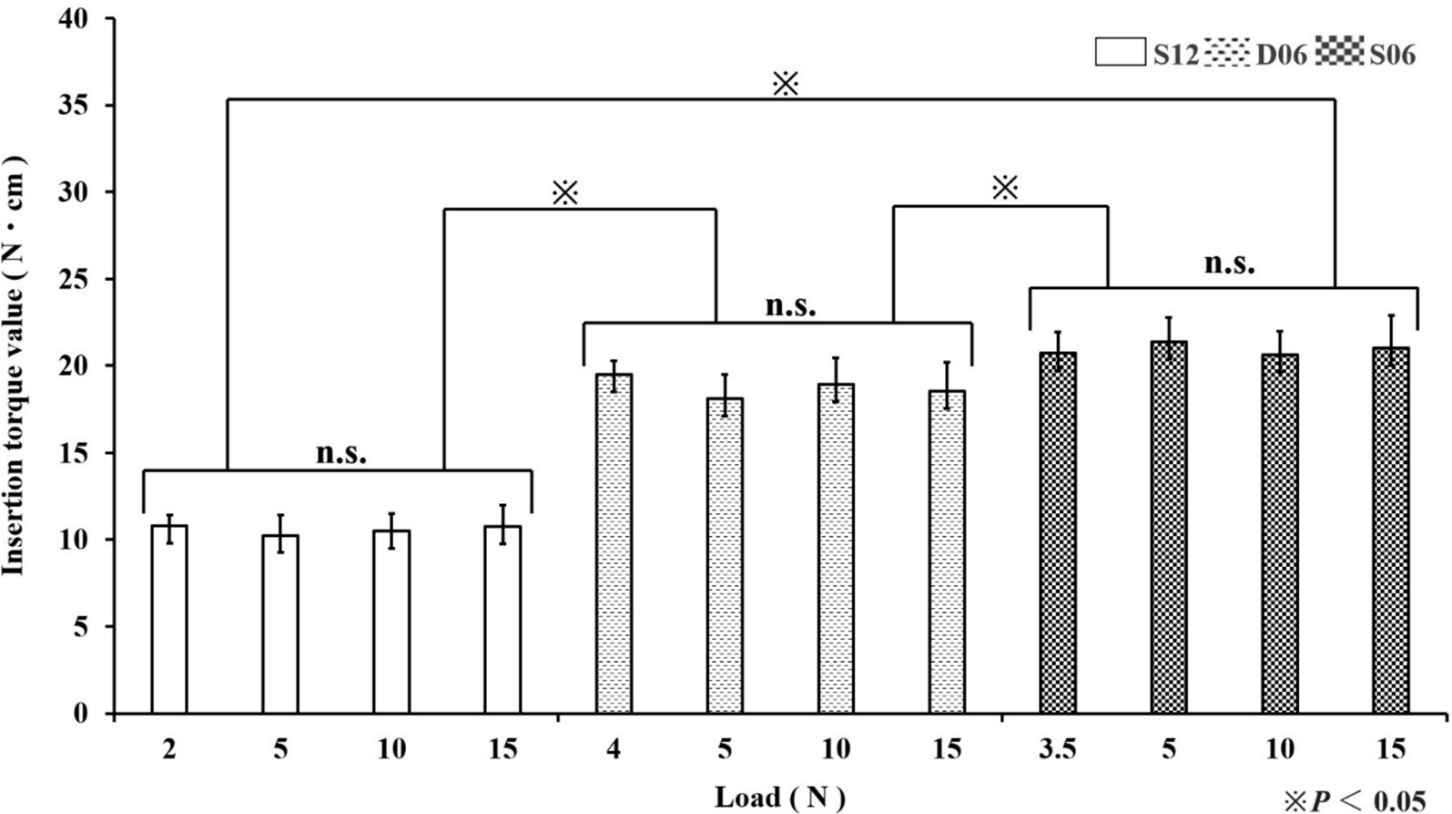
ITV at different insertion loads

**Fig. 6:**
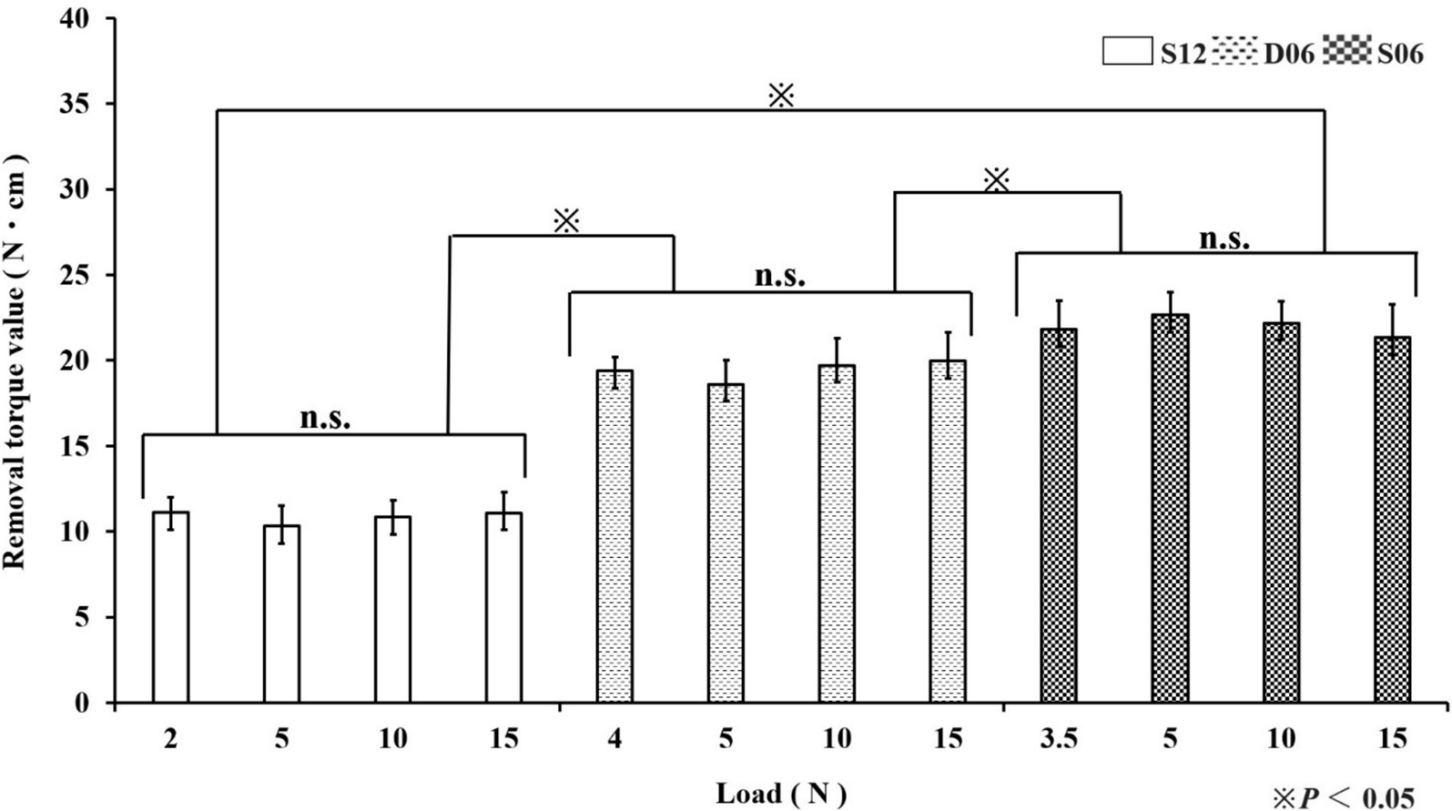
RTV at different insertion loads

For both the ITV and RTV, across all the loads (minimum insertion load, 5.0 N, 10.0 N, and 15.0 N), S06 presented the highest values, followed by D06 and then S12, with significant differences observed among the implant types. In contrast, no significant differences were observed among the various insertion loads within each implant type or between the ITV and RTV (*p* < 0.05) (Figs. 5 and 6).

### Pattern analysis of the insertion time

**Fig. 7:**
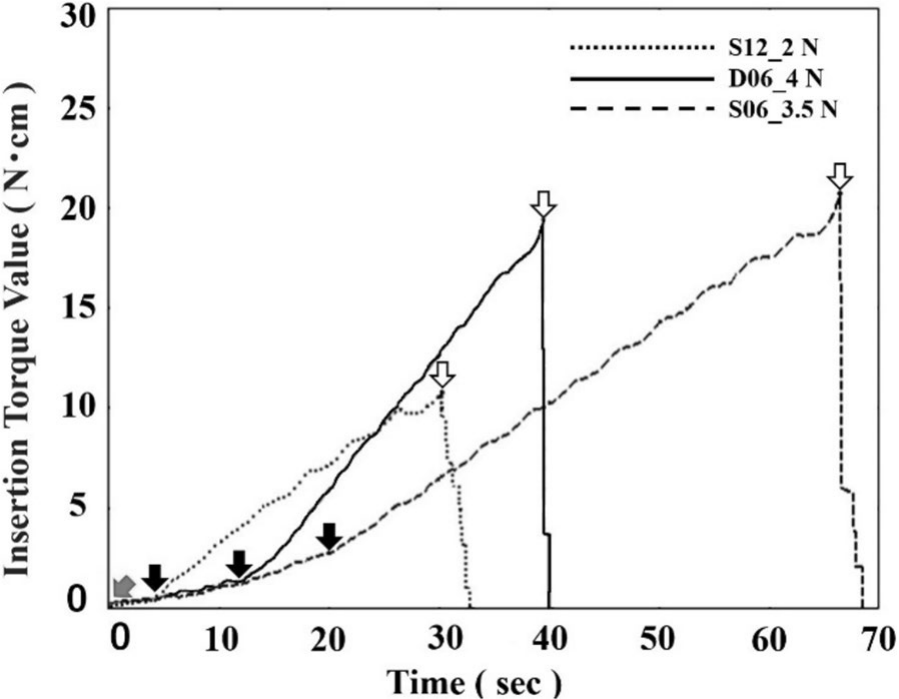
Pattern analysis of the insertion torque–time curve for minimizing the insertion load. The figure illustrates the torque–time curves of three types of implants as an example under minimum insertion load conditions. The figure shows the initial insertion torque point (red arrow), the insertion-starting point (black arrows), and the insertion endpoint (white arrows). From the initial insertion torque point to the insertion-starting point, the curve represents the free-spinning zone, during which the implant does not engage with the prepared site, resulting in no increase in the insertion torque, indicating a free-spinning state. From the insertion starting point to the insertion endpoint, the curve represents the constant insertion zone, where the implant is steadily inserted into the prepared site, and the insertion torque consistently increases. The insertion process is completed at the insertion endpoint, and the time from the initial insertion torque point to the insertion endpoint is defined as the insertion time

**Fig. 8:**
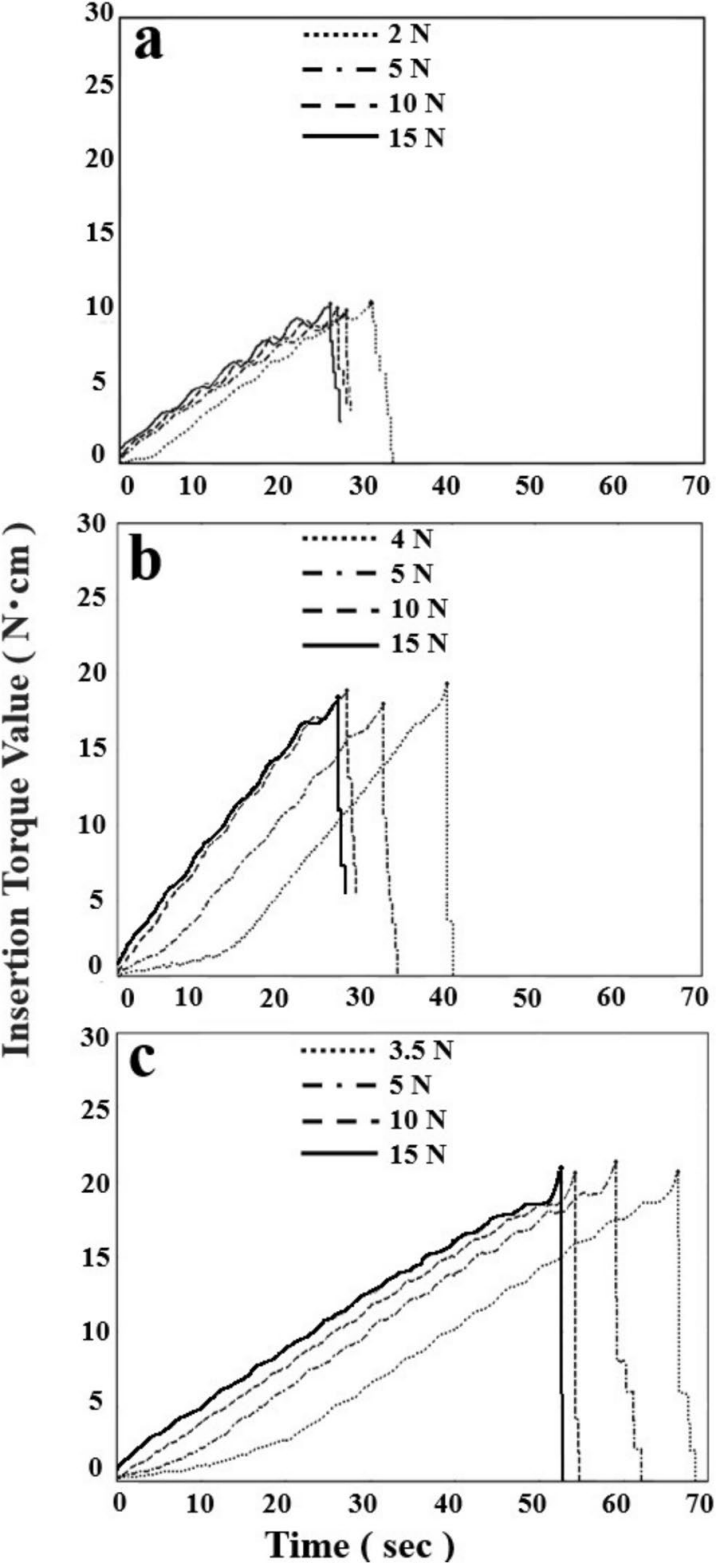
The torque‒time curves for different insertion loads at SI2, D06 and S06. **a**: S12, (**b**): D06, (**c**): S06

The recorded torque–time curves can be categorized as shown in Fig. 7. For all the implants, as the insertion load decreased, the torque–time curves shifted to the right (Figs. 8a, b, and c). For S12, the slope of the linear portion of the torque–time curve was consistently approximately 0.36–0.39 N cm/sec regardless of the insertion load. However, the time taken for the torque to begin rising linearly varied. At the minimum insertion load of 2.0 N, a delay of 5 s was observed relative to the other loads (Table 1). The insertion time, which is the time required to reach the maximum torque value, was the longest at an insertion load of 15.0 N, followed by 10.0 N, 5.0 N, and then the minimum insertion load of 2.0 N (Fig. 8a).

**Table 1:**
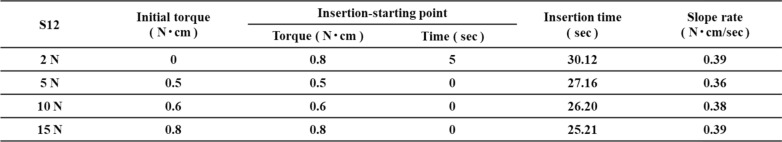
Analysis of the insertion time for each insertion load in S12

For D06, similar to S12, the torque–time curves shifted to the right with decreasing insertion load (Fig. 8b). The slope of the linear portion was 0.63–0.68 N cm/sec. Differences in the time required for the torque to rise linearly were observed. A delay of 14 s was noted at the minimum insertion load of 4.0 N, and a delay of 6 s was noted at an insertion load of 5.0 N (Table 2). The trend in the insertion time for D06 was similar to that observed for S12, with longer times at 15.0 N, followed by 10.0 N, 5.0 N, and then 4.0 N.

**Table 2:**
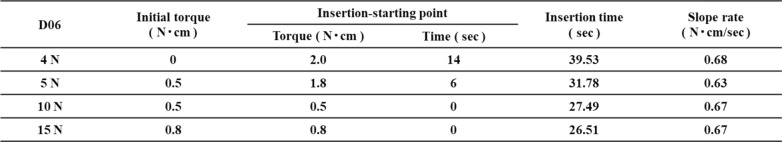
Analysis of the insertion time for each insertion load in D06

For S06, the slope of the linear portion was similar to that of S12 (approximately 0.37–0.39 N cm/sec). Additionally, a shift of 20 s was observed at an insertion load of 3.5 N, and 9 s was observed at 5.0 N (Table 3). The insertion time for S06 followed the same trend, being the longest at 15.0 N, followed by 10.0 N, 5.0 N, and finally 3.5 N (Fig. 8c).

**Table 3:**
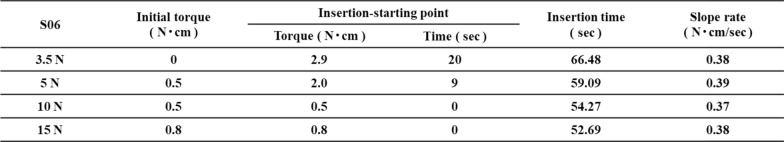
Analysis of the insertion time for each insertion load in S06

When the insertion load was varied from the minimum insertion load to 15.0 N, the overall shape of the torque–time curves differed depending on the implant design. However, the slopes of the linear portions remained unchanged. A rightward shift of the torque–time curves was observed at lower insertion loads, with maximum time shifts of 5 s for S12, 14 s for D06, and 20 s for S06. Furthermore, in all three implant types, increasing the insertion load was associated with a higher initial insertion torque value, which is defined as the torque value observed immediately after insertion.

### Evaluation of artificial bone and implant contact

**Fig. 9:**
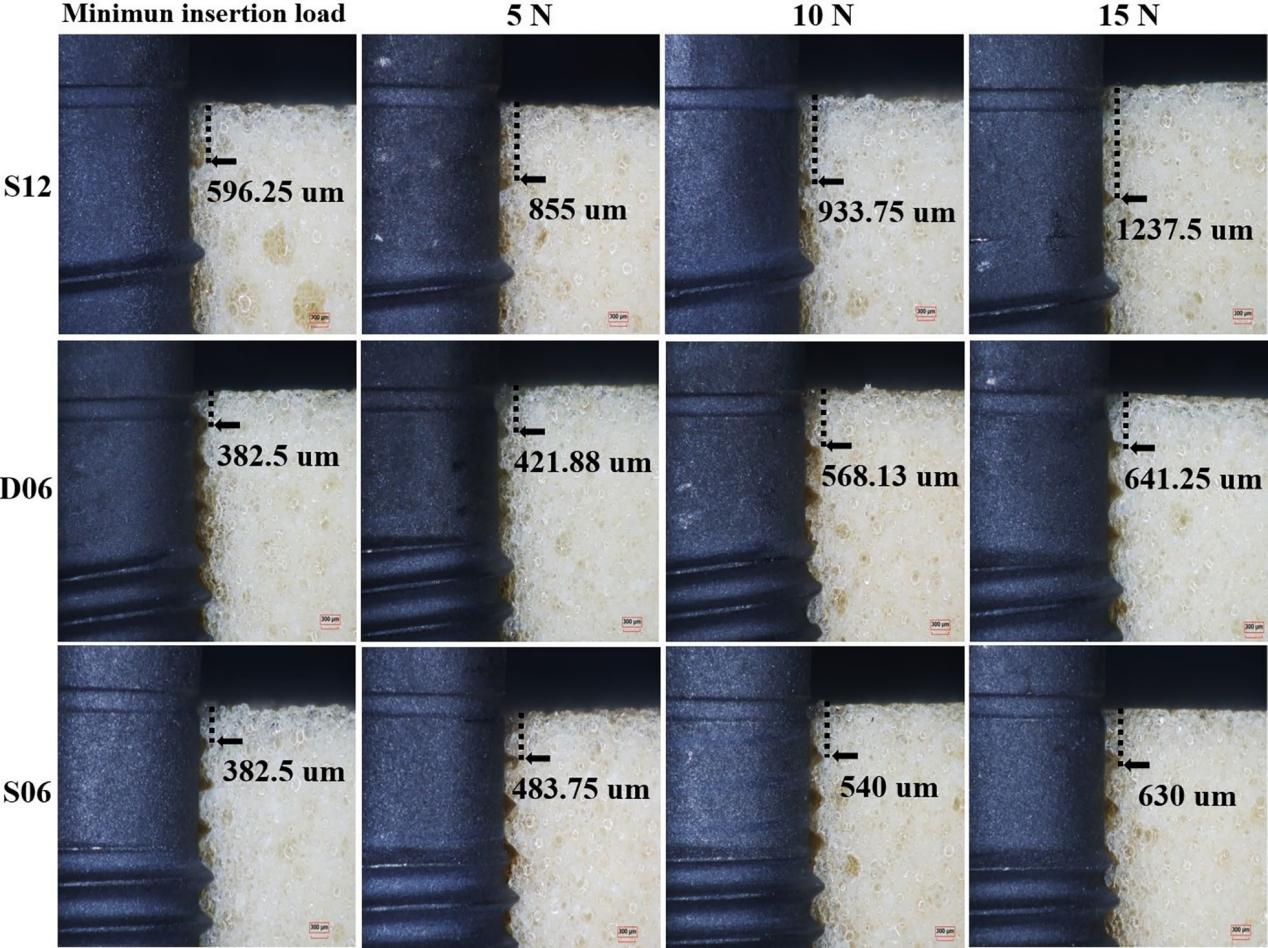
Artificial bone‒implant interface at the upper region of the implants under various insertion loads (magnification, × 50). This figure illustrates the bone‒implant interface at the upper region of the implants (S12, D06, S06) under different insertion load conditions: minimum insertion load, 5 N, 10 N, and 15 N. The minimum insertion load for each implant is as follows: S12: 2.0 N, D06: 4.0 N, S06: 3.5 N. The arrows indicate the voids at the first thread that passed during implant insertion. The values (e.g., 596.25 μm, 421.88 μm) represent the depth of the contact area at the bone–implant interface under each load condition

**Fig. 10:**
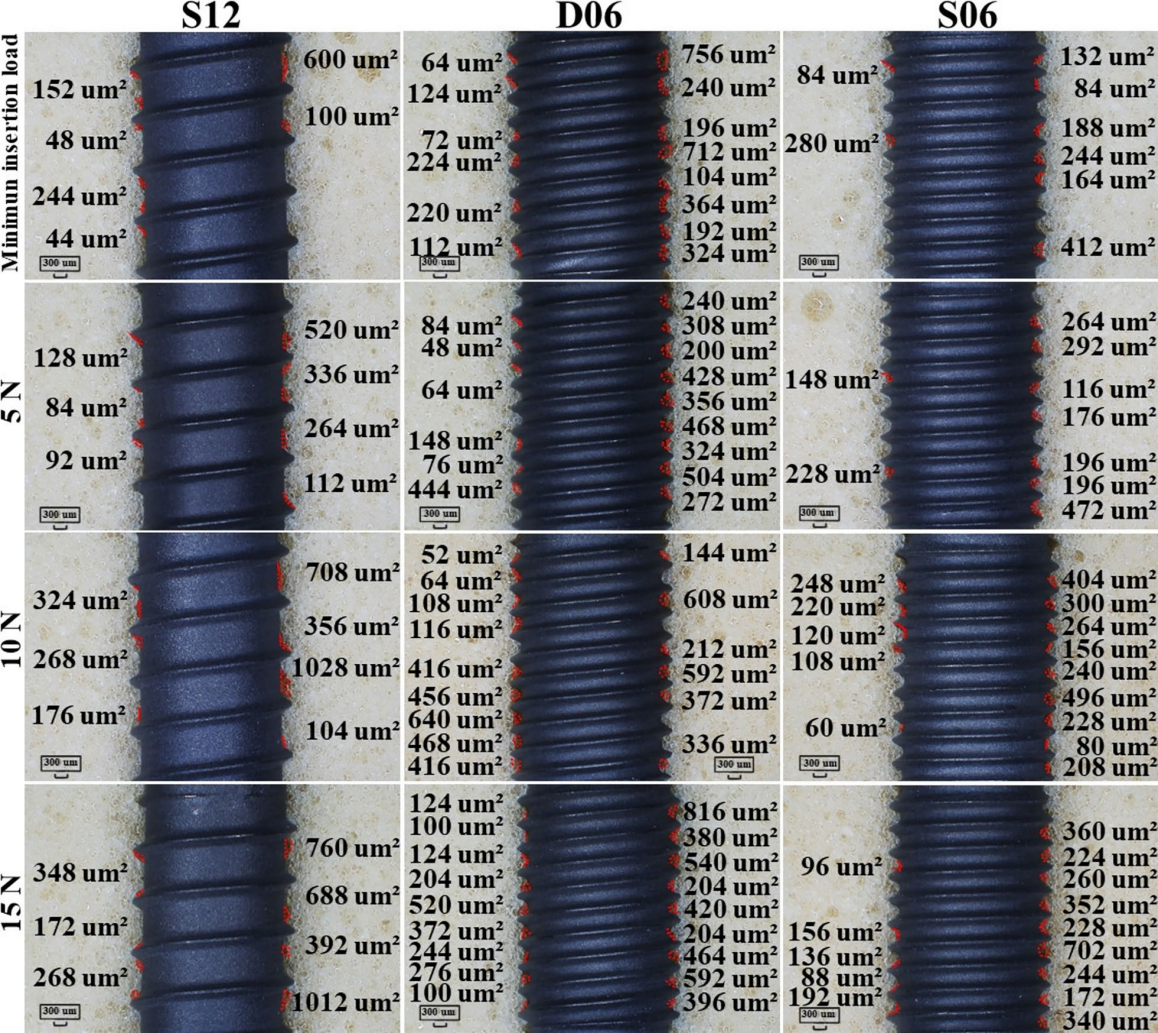
Void area at the bone‒implant interface for each implant and insertion load (magnification, × 20). This figure shows the void area at the bone‒implant interface for three implant types (S12, D06, and S06) under various insertion load conditions: minimum insertion load, 5 N, 10 N, and 15 N. The minimum insertion load for each implant is as follows: S12: 2.0 N; D06: 4.0 N; and S06: 3.5 N. The values displayed in the red area (e.g., 152 μm^2^, 520 μm^2^) represent the measured void area for each thread at the bone–implant interface under the respective load conditions

**Fig. 11:**
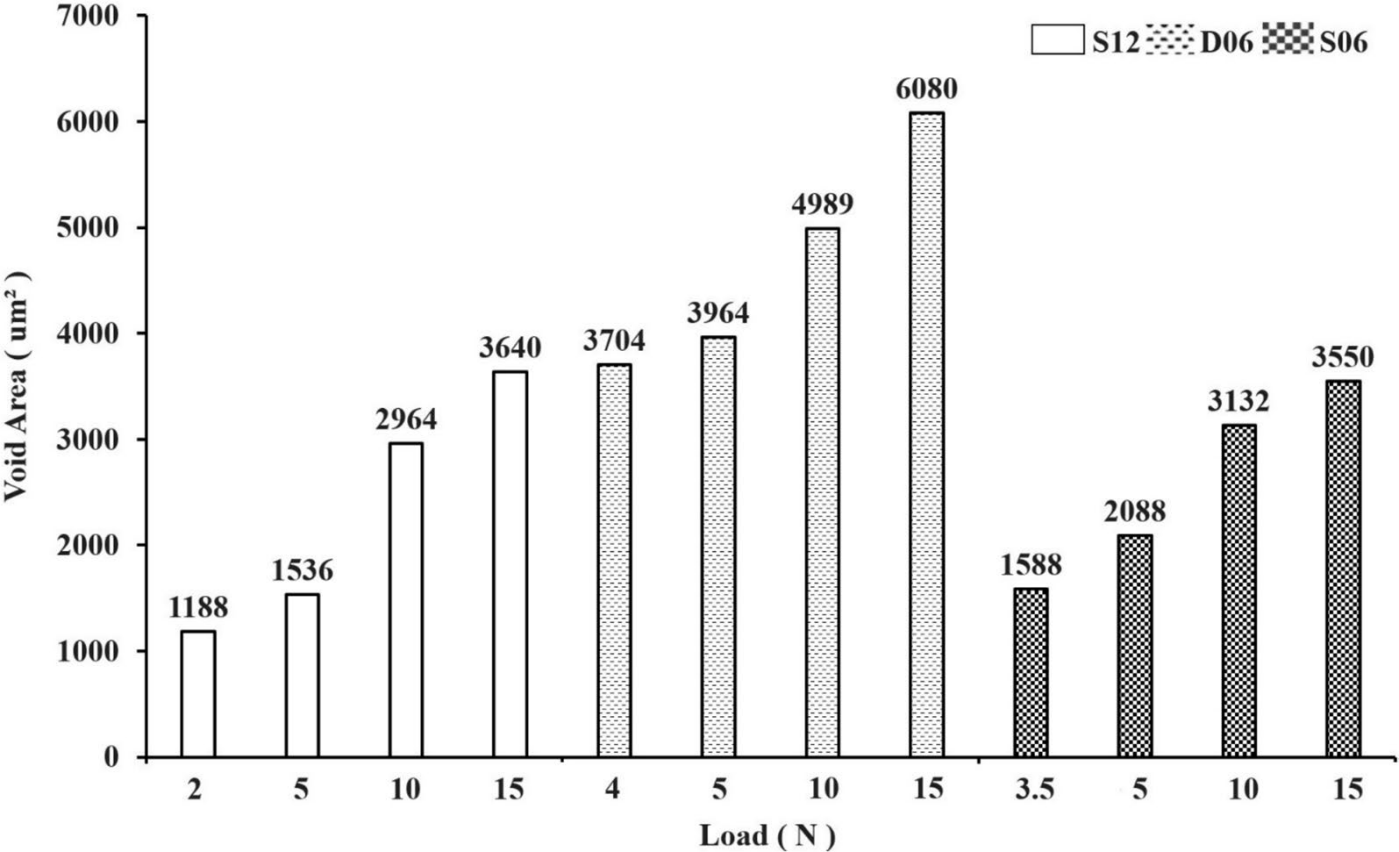
Total void area at the bone‒implant interface under various insertion loads for different implant types

At the contact interface between the coronal portion of the implant and the artificial bone, cavities created by the passage of the leading thread were observed (Fig. 9). These cavities indicate the position at which the thread tip engaged the artificial bone, and with increasing insertion load, the cavity was observed to shift further in the apical direction. At the minimum insertion load, the depth of thread engagement corresponded to approximately half the lead of each implant (Fig. 9). At an insertion load of 15.0 N, the void created by the passage of the first thread was located deeper than the lead length of the implant. At the interface between the implant body and the artificial bone, voids, which are located primarily in the thread troughs, resulting from compression-induced fracturing and the subsequent displacement of artificial bone fragments, were observed. The number of voids measured at the minimum insertion load, 5.0 N, 10.0 N, and 15.0 N, was as follows: for S12, 6, 7, 7, and 7; for D06, 14, 15, 15, and 18; and for S06, 8, 9, 14, and 14 (Fig. 10). The total void area increased proportionally with the insertion load (Fig. 11), and the void area for D06 increased to a greater extent than those for S12 and S06. Furthermore, the rate of increase in void area when the insertion load was increased from 5.0 N to 10.0 N was greatest at 48.2% for S12, 21.0% for D06, and 33.3% for S06.

## Discussion

### Effect of the insertion load

In clinical practice, parameters such as the ITV and implant stability quotient (ISQ) are used to assess primary stability [13, 14]. ITV is reportedly more sensitive than ISQ is [14, 16]. Although the RTV, which measures the torque required to loosen an inserted implant, is a destructive parameter and is therefore not used clinically, it is employed in basic research using ex vivo models such as artificial bone [16, 17]. In the present study, RTV was utilized to evaluate whether the stability indicated by the ITV, a more sensitive measure of primary stability than the ISQ, was maintained until implant removal following the application of the insertion load. The results demonstrated that both the ITV and RTV were highest for S06, followed by D06 and then S12, indicating that these parameters are influenced by the implant design [16]. Conversely, variations in the insertion load did not affect either the ITV or RTV, a finding that is both clinically advantageous and significant in terms of ensuring the stability of the insertion procedure.

### Mechanism of minimum insertion load

The minimum insertion load was influenced by the pitch, lead angle, and number of threads of each implant. The lead angle, which is defined as the angle at which the thread tip engages the bone [16], requires a higher insertion load for S06, whose lead angle is half that of S12, even though both are single-thread designs. Furthermore, although D06 shares the same lead angle as S12 does, there is a need to simultaneously engage two threads. The characteristic of a double-thread design resulted in D06 requiring the highest minimum insertion load.

### Effect of the insertion time

One of the most notable changes observed when the insertion load was varied was the change in the time required to reach the maximum torque value, represented as the insertion time. Although the insertion time is determined by the guidance provided by the threads, the present study suggests that lower insertion loads, rather than higher loads leading to shorter insertion times, result in prolonged insertion times. Given that the implant rotates at a constant speed, it is presumed that a lower insertion load increases the likelihood of slippage, thereby extending the insertion time.

### State of the implant and artificial bone interface

During implant insertion, compressive forces along the long axis of the implant generate shear stresses in the regions where the implant contacts the implant bed wall [20]. When the load was increased from 5.0 N to 10.0 N, the void ratio at the interface reached a maximum. Moreover, at an insertion load of 15.0 N, the implant engaged with the artificial bone beyond the length of the lead, suggesting that insertion loads exceeding 10.0 N may have a detrimental effect on the surrounding tissues. In particular, the multithread D06, with its larger lead angle and small pitch of 0.6 mm, was presumed to induce greater damage at the implant–artificial bone interface. Under the present experimental conditions, while damage at the interface did not affect the ITV or RTV, its potential impact on secondary fixation remains unclear and requires further investigation.

### Optimal insertion load

One potential optimal or recommended insertion load is one that allows the thread to engage to a depth of approximately half of its lead. Clinically, considering the variability in the minimum insertion load, it is presumed that an insertion load slightly higher than the minimum would be preferable. Furthermore, a relatively low insertion load that prevents slippage was associated with minimal damage at the implant–artificial bone interface; thus, maintaining a minimal insertion load once the thread has engaged the bone may help avoid damage to the surrounding tissues. In this study, we employed artificial bone models replicating the poor bone quality of the posterior maxilla, where obtaining primary stability is known to be challenging, to rigorously evaluate how varying insertion loads and implant macro-designs perform under high biological and mechanical risk. Our findings indicate that the insertion load within a certain range does not directly affect primary stability metrics (ITV, RTV) and that insertion loads below the minimum insertion load prolong the implant insertion time and compromise surgical efficiency, while excessive loads cause void from bone fragment loss and increase the risk of bone damage and remodeling. These results help define a clinically acceptable insertion load, which is especially critical in areas of low bone quality. However, caution must be exercised when directly extrapolating the results obtained in poor-quality bone to the mandible. The mandible is generally classified as type I to type II bone quality, in which the same insertion load achieves greater primary stability than in the maxilla and is more susceptible to damage from overloading. Currently, insertion load management depends on surgeon experience rather than quantitative standards. Therefore, future efforts should focus on connecting the implant design and insertion load to each patient’s bone quality. To achieve this goal, guidelines and tools that enable quantitative measurement and control of the insertion load are needed. For example, employing navigation systems with real-time load feedback functionality could reduce the variability of operators and enable reproducible, high-precision implant placement. At present, however, controlling the insertion load in clinical practice remains challenging, and developing methods for its precise regulation is an important subject for future research. In 2017, an implant robot (YOMI, Neocis Inc., Miami, Florida, USA) received FDA approval for dental applications [21]. With the recent emergence of medical robots capable of precisely controlling forces via load cells [22], even implant insertion procedures may benefit from a level of precision beyond the capabilities of human tactile feedback.

A limitation of this study is that it was conducted under restricted conditions using prototype implants and artificial bone; thus, the present results represent only a portion of the potential indicators of the insertion load. Further studies incorporating a wider range of conditions, such as variations in the density of the artificial bone blocks and different implant design combinations, are warranted.

## Conclusions

This study conducted simulation experiments using prototype implants and artificial bone to elucidate the impact of the insertion load on primary stability. It was demonstrated that a minimum insertion load exists for implant insertion and that the required insertion load differs according to the implant design. The minimum insertion load was influenced by the lead angle and the number of threads, with significant differences observed in the order S12 < S06 < D06. Furthermore, varying the insertion load from the minimum to 15.0 N did not affect either the ITV or RTV. However, higher insertion loads resulted in increased damage at the implant–artificial bone interface, with D06 exhibiting notably greater interface destruction than S12 and S06.

## Data Availability

No datasets were generated or analysed during the current study.

## Abbreviations

ITV: Insertion torque value
RTV: Removal torque value
